# Endoplasmic reticulum stress promoted acinar cell necroptosis in acute pancreatitis through cathepsinB-mediated AP-1 activation

**DOI:** 10.3389/fimmu.2022.968639

**Published:** 2022-08-19

**Authors:** Xiao Han, Bin Li, Jingpiao Bao, Zengkai Wu, Congying Chen, Jianbo Ni, Jie Shen, Pengli Song, Qi Peng, Rong Wan, Xingpeng Wang, Jianghong Wu, Guoyong Hu

**Affiliations:** ^1^ Department of Gastroenterology, Shanghai General Hospital, Shanghai Jiao Tong University School of Medicine, Shanghai, China; ^2^ Shanghai key Laboratory of Pancreatic Disease, Institute of Pancreatic Disease, Shanghai Jiao Tong University School of Medicine, Shanghai, China

**Keywords:** acute pancreatitis, endoplasmic reticulum stress, necroptosis, cathepsin B, activating protein-1

## Abstract

Acinar cell death and inflammatory response are two important events which determine the severity of acute pancreatitis (AP). Endoplasmic reticulum (ER) stress and necroptosis are involved in this process, but the relationships between them remain unknown. Here, we analyzed the interaction between ER stress and necroptosis and the underlying mechanisms during AP. Experimental pancreatitis was induced in Balb/C mice by caerulein (Cae) and lipopolysaccharide (LPS) or L-arginine (L-Arg) *in vivo*, and pancreatic acinar cells were also used to follow cellular mechanisms during cholecystokinin (CCK) stimulation *in vitro*. AP severity was assessed by serum amylase, lipase levels and histological examination. Changes in ER stress, trypsinogen activation and necroptosis levels were analyzed by western blotting, enzyme-linked immunosorbent assay (ELISA), adenosine triphosphate (ATP) analysis or lactate dehydrogenase (LDH) assay. The protein kinase C (PKC)α -mitogen-activated protein kinase (MAPK) -cJun pathway and cathepsin B (CTSB) activation were evaluated by western blotting. Activating protein 1 (AP-1) binding activity was detected by electrophoretic mobility shift assay (EMSA). We found that ER stress is initiated before necroptosis in CCK-stimulated acinar cells *in vitro*. Inhibition of ER stress by 4-phenylbutyrate (4-PBA) can significantly alleviate AP severity both in two AP models *in vivo*. 4-PBA markedly inhibited ER stress and necroptosis of pancreatic acinar cells both *in vitro* and *in vivo*. Mechanistically, we found that 4-PBA significantly reduced CTSB maturation and PKCα-JNK-cJun pathway -mediated AP-1 activation during AP. Besides, CTSB inhibitor CA074Me markedly blocked PKCα-JNK-cJun pathway -mediated AP-1 activation and necroptosis in AP. However, pharmacologic inhibition of trypsin activity with benzamidine hydrochloride had no effect on PKCα-JNK-cJun pathway and necroptosis in CCK-stimulated pancreatic acinar cells. Furthermore, SR11302, the inhibitor of AP-1, significantly lowered tumor necrosis factor (TNF) α levels, and its subsequent receptor interacting protein kinases (RIP)3 and phosphorylated mixed lineagekinase domain-like (pMLKL) levels, ATP depletion and LDH release rate in CCK-stimulated pancreatic acinar cells. To sum up, all the results indicated that during AP, ER stress promoted pancreatic acinar cell necroptosis through CTSB maturation, thus induced AP-1 activation and TNFα secretion *via* PKCα-JNK-cJun pathway, not related with trypsin activity. These findings provided potential therapeutic target and treatment strategies for AP or other cell death-related diseases.

## Introduction

Acute pancreatitis (AP), the most common diseases of pancreas in the globe, sometimes is lethal. But the pathogenic mechanisms have not been fully elucidated, and no effective treatment is available (1–3). Pancreatic necrosis is an important cause of AP worsening and patient’s death (4, 5). Necrosis has long been considered as a haphazard or passive event, but found to be regulated as well. Programmed necrosis appears as necroptosis, pyroptosis, ferroptosis or other types (6–9). Among them, necroptosis is the best investigated form, which involves the activation of receptor interacting protein kinases (RIP) 3 - mixed lineage kinase domain-like (MLKL) pathway (6, 7). He’s team and Zhang’s team found almost at the same time that RIP3 deletion improved experimental AP in mice (6, 7). Besides, we reported the imbalance between RIP1 and RIP3 shifted cell death to necrosis in our previous studies, which unraveled that necroptosis promotes the development of AP (10). But the underlying mechanisms of necroptosis remain unclear in AP.

Endoplasmic reticulum (ER) stress activation is an early event during experimental AP, and inhibition of ER stress obviously alleviates pancreatic injury (11–14). In response to ER stress, three proteins are activated, including activating transcription factor 6 (ATF6), inositol-requiring ER-to-nucleussignal kinase 1 (IRE1) and protein kinase-like ER kinase (PERK), then leads to expression of glucose-related peptide 78 (GPR78), small intron from X-box–binding protein 1 (sXBP1), C/EBP homology protein (CHOP) and so on (13, 15). Previous studies have demonstrated that inhibition of ER stress by 4-phenylbutyric acid prevented vital organ injury and intestinal epithelial cell apoptosis in rats with AP (16, 17). Although ER stress is widely reported to be associated with apoptosis, recent studies have revealed that it can trigger necroptosis in L929 cells, microglia/macrophages or hepatocyte (18–20). However, the regulatory role of ER stress on necroptosis of pancreatic acinar cells and its mechanisms need to be further explored.

Intrapancreatic trypsinogen activation is an important initiating event of AP, during which, cathepsin B (CTSB) cleaved trypsinogen to mature trypsin and released its NH2-terminal trypsinogen activating peptide (TAP), an indicator of trypsinogen activation (21). Therefore, necroptosis, ER stress and CTSB-induced trypsinogen activation are all involved in AP (14). Their relationship is so complex that it needs to be clarified during AP. On the one hand, activation of ER stress increased CTSB activity, while inhibition of ER stress decreased it in isolated pancreatic acinar cells in AP (22). On the other hand, excessive CTSB released from lysosomes into the cytosol can convert the cell death pathway to necrosis during AP (23). Therefore, CTSB may serve as a key molecule mediating trypsin-induced necrosis and ER stress-induced necroptosis. To illuminate the role and mechanisms of CTSB in AP are of great significance for further elucidating the pathogenesis of AP. Generally, necroptosis is started by engagement of death receptors with the ligands, such as tumor necrosis factor-α (TNFα) (24), which is encoded by transcription factors, for example, nuclear factor-kappa B (NF-κB) and activating protein-1 (AP-1) (25, 26). Besides, ER stress can induce the synthesis of inflammatory cytokines through these transcription factors (20, 24). However, as is reported, necroptosis blockade by RIP3 siRNA had no effect on trypsinogen activation (10). Furthermore, previous studies showed that activation of protein kinase C (PKC), mitogen-activated protein kinases (MAPKs) and -AP-1, subsequently induced autocrine production of TNFa and cell necroptosis (24). Therefore, in present study, we intervened ER stress, CTSB, trypsin or AP-1 respectively, then investigated the effects of ER stress on necroptosis during AP and its specific mechanisms.

## Materials and methods

### Reagents

Caerulein (Cae; cat # C9026), L-arginine (L-Arg; cat # A5131), lipopolysaccharide (LPS; cat # L2880), cholecystokinin 8 (CCK 8, cat # C2175), 4-phenylbutyrate (4-PBA; cat # SML0309), and benzamidine hydrochloride (Ben; cat # 434760) were purchased from Sigma-Aldrich Chemical (St. Louis, MO, USA). CA074 Methyl ester (CA074Me, cat # S7420) was purchased from Selleck Chemicals (Houston, TX, USA). SR11302 (cat # 160162-42-5) was purchased from APExBIO (Houston, TX, USA). Antibodies against X-box–binding protein 1 (sXBP1, cat # sc-7160) and receptor interacting protein kinase 3 (RIP3, cat # sc-135171) were from Santa Cruz Biotechnology (Dallas, TX, USA). Antibodies against C/EBP homology protein (CHOP, cat # 2895), cathepsin B (CTSB, cat # 31718), phosphorylated protein kinase α (p-PKCα, cat # 9375), phosphorylated c-Jun NH2-terminal kinase (p-JNK, cat # 4668), phosphorylated extracellular signal-regulated kinas (p-ERK, cat # 4370), phosphorylated p38 mitogen activated protein kinases (p-p38MAPK, cat # 4511), phosphorylated cJun (p-cJun, cat # 3270) and IL1β(3A6) (cat # 12242) were from Cell Signaling Technology (Danvers, MA, USA). Antibodies against mixed lineage kinase domain-like (MLKL, cat # ab172868), phosphorylated mixed lineagekinase domain-like (pMLKL, cat # ab196436) and Ly6G (cat # ab25377) were from Abcam (Cambridge, MA, USA). Antibodies against glucose-related peptide 78 (GPR78, cat # 11587-1-AP) and TNFα (cat # 60291-1-Ig) were from Proteintech Biotechnology (Wuhan, China). Antibody against IL6 (cat # BS6419) was purchased from Bioworld Technology(St. Louis Park, MN, USA),and antibody against β-actin (cat # AF0003) was from Beyotime Biotechnology (Shanghai, China). Nuclear and cytoplasmic protein extraction kit was from Pierce (Rockford, IL, USA). The biotin-labeled probe containing the activating protein 1 (AP-1) binding site was purchased from Beyotime Biotechnology (Shanghai, China). The light shift chemilumines-cent EMSA kit was from Pierce (Rockford, IL, USA).

### Mouse strains

Balb/C mice (6 ~8 weeks, 20~22g, male) were purchased from Shanghai SLAC Laboratory Animal Co Ltd (Shanghai, China). All mice were kept in pathogen-free conditions in individually-ventilated cages (4 ~6 mice per cage) at 23 ± 2°C and a 12 h dark/light cycle with free access to water and standard rodent diet before experiment. All mice were allocated into groups in a completely randomized manner (n = 6 per group) to conduct the experiment. All experiments were approved by the Animal Ethics Committee of Shanghai Jiao Tong University School of Medicine (SYXK 2013-0050, Shanghai, China.).

### Induction of experimental AP and treatments

Two AP models were built *in vivo*, both are widely used, rapidly induced and noninvasive (27). One is induced by injections of caerulein (100 μg/kg, i.p. with 1 h interval between injections, ten injections) and LPS (5 mg/kg, i.p. administered immediately after the last injection of caerulein) as reported before (28, 29). Controls received normal saline (NS) equivalent to caerulein. The first caerulein injection was defined as 0 h. The ER stress inhibitor (4-PBA, 4mg per mouse) or the CTSB inhibitor (CA074Me, 10 mg/kg) was injected intraperitoneally (i.p.) 0.5 h before the first caerulein injection and mice were sacrificed at 12 h. The other model is induced by L-Arg (4 g/kg, 8%, pH=7.0, with 1 h interval between injections, two injections) as previously described (30–33). Control mice received equal NS instead of L-Arg. The second L-Arg injection is defined as day 0. 4-PBA (4mg per mouse) was injected 0.5 h i.p. before the first L-Arg injection, and equivalent 4-PBA was added everyday in the following two days. Mice were sacrificed at day 3. Serum and pancreas were collected. Histological scoring of haematoxylin and eosin (H&E) sections were performed by two experienced pathologists (32–34).

### Serological test

Blood samples of each group were collected and centrifuged at 250 g for 20 min at 4°C. Serum amylase and lipase activities were measured by enzyme dynamics chemistry, according to the manufacturer’s instructions in a Roche/Hitachi Modular Analytics System (Roche, Basel, Switzerland).

### Haematoxylin and eosin and immunohistochemical staining

Mice pancreas specimens were fixed in 4% neutral paraformaldehyde for 24 ~48 h, embedded in paraffin, and cut into 4 μm sections for H&E staining by standard procedures. Endogenous peroxidase was neutralized by 3% hydrogen peroxide. Then sections were incubated overnight at 4°C with monoclonal antibody against Ly6G (1:100). After being rinsed in PBS for three times, sections were incubated with secondary antibody for 1 h at 37°C and then imaged by an ultrasensitive SP kit and a DAB kit (Fuzhou Maxin, China). Pancreatic tissue section were scored on a range of 0 to 3 (0 represented normal appearance and 3 meant severe), based on the presence of necrosis, edema and inflammation (35). The pathologists were blinded to the experiment groups.

### Pancreatic acinar cell isolation and *in vitro* cultures

Pancreatic acinar cells were isolated from Balb/C mice as described previously, using collagenase digestion with minor modifications (32, 33, 36). Primary pancreatic acinar cells were incubated at 37°C in Dulbecco’s modified Eagle’s medium/Ham (DMEM) F-12 medium containing 10% fetal bovine serum (FBS). Acinar cells were pre-treated with ER stress inhibitor 4-PBA (2.5 μM, 5 μM, 10 μM) or CTSB inhibitor (CA074Me; 50 μM) or trypsin inhibitor Ben (1 μM) or AP-1 inhibitor SR11302 (10 μM) for 30 min, and then stimulated by 200 nM CCK 8 for 30 min or 6 h. Cells were collected at the time points as indicated in the Figure legends.

### Western blotting

Pancreatic tissue and pancreatic acinar cell extracts were used for western blotting analysis. Total amounts of protein were detected using the bicinchoninic acid assay method (Beyotime Biotechnology, China). Proteins (40 μg per lane) were separated by 10% SDS-PAGE at 120V and transferred to nitrocellulose membranes (Millipore, Mass, USA) for 30 ~ 60 min. Membranes were then incubated with primary antibodies against polyclonal GRP78 (1:1000), sXBP1 (1:400), RIP3 (1:400), MLKL (1:1000), IL-1β (1:1000), IL-6 (1:1000) and p-PKCα (1:400); monoclonal CHOP (1:1000), pMLKL (1:1000), CTSB (1:1000), p-JNK (1:1000), p-ERK (1:1000), p-p38MAPK (1:1000), p-cJun (1:1000), TNFα (1:1000) and β-actin (1:1000) overnight at 4°C. After being washed with PBS containing 0.1% Tween, membranes were probed by secondary antisera labelled with goat anti-mouse or goat anti-rabbit IR-Dye 700 or 800 cw for 1 h at 37°C. Membranes were scanned by an Odyssey Infra-red Scanner (LI-COR, Lincoln, NE, USA). Representative blot images were presented from three separate experiments. Relative expression of target proteins was expressed as fold changes compared to normal control after normalized to β-actin.

### ATP analysis

Cell survival assay was performed according to the manufacturer’s instructions of Cell Titer-GloLuminescent Cell Viability Assay kit (Promega, Madison,WI). Briefly, 50 μL ATP detection reagents were added into 100 μL cell suspension in a 96-well culture plate, and the levels of bioluminescence were recorded using a SpectraMax 190 system (Molecular Devices, San Jose, CA, USA).

### Lactate dehydrogenase assay

Quantitative analysis of cytotoxicity can be achieved by detecting the activity of LDH released into the culture supernatant from injured cells using LDH Cytotoxicity Assay Kit (Beyotime Biotechnology, Shanghai, China) according to the manufacturer’s instructions. Briefly, pancreatic acinar cells in each group were collected and centrifuged at 100 g for 5 min at 4°C. A triplicate set of wells for the culture medium background with no cells were served as minimum (blank LDH), and untreated normal control cells lysed to yield LDH completely were used for maximum (total LDH), respectively. Then 120 μL of supernatant and 60 μL of LDH detection reagent were mixed in a 96-well plate for 30 min. A SpectraMax 190 system (Molecular Devices, San Jose, CA, USA) was used to detect the absorbance at 490 nm. The LDH release rate was calculated according to the following formula: LDH release rate (%) = [(sample LDH – blank LDH)/(total LDH – blank LDH)] × 100%.

### Measurement of activated trypsin

Trypsinogen activation peptide (TAP) is a small peptide released from trypsinogen during its activation (37). Therefore, TAP concentration can indirectly reflect the levels of trypsinogen activation. TAP levels of pancreatic acinar cells were measured by enzyme-linked immunosorbent assay (ELISA) according to the manufacturer’s protocols (Westang Bio-Tech, Shanghai, China).

### Electrophoretic mobility shift assay

The binding activity of AP-1 was detected by EMSA. A biotin-labelled probe containing AP-1 binding site was incubated with 10 μg nuclear extracts for 30 min at room temperature. Protein-DNA complexes were separated by 5% polyacrylamide gel in 0.5x Tris Buffer EDTA at first and then transferred to a positively charged nylon membrane. The biotin-labelled DNA was examined by a chemiluminescent detection kit.

### Statistical analysis

All the data are presented as Mean ± SEM, from at least three replicates with six mice per group. The distribution of data was assessed by Kolmogorov–Smirnov test at first. If data followed a Gaussian distribution, parametric tests were carried out: Student’s t test for two groups and one-way ANOVA for three or more groups. If data were not normally distributed, non-parametric tests were performed: Mann–Whitney test for two groups and Kruskal–Wallis test for three or more groups. All analyses were performed using GraphPadPrism (version 7.00 for Windows, GraphPad Software, La Jolla California USA, www.graphpad.com). *p* value <0.05 was considered as statistically significant.

## Results

### ER stress was initiated before necroptosis in CCK-stimulated pancreatic acinar cells

**Figure 1:**
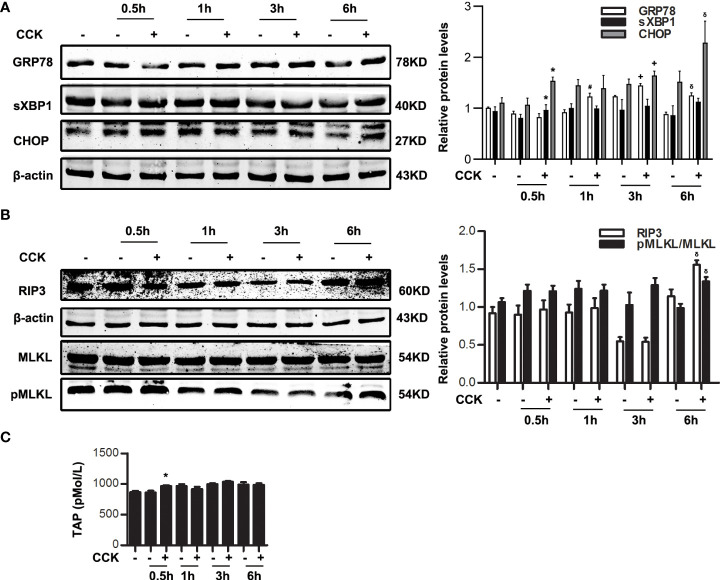
ER stress was initiated before necroptosis in CCK-stimulated pancreatic acinar cells. Pancreatic acinar cells were stimulated by 200 nM CCK for 0.5 h, 1 h, 3 h and 6 h. **(A)** Immunoblot analysis of GRP78, sXBP1 and CHOP levels in pancreatic acinar cells. **(B)** Immunoblot analysis of RIP3, MLKL and pMLKL levels in pancreatic acinar cells. **(C)** ELISA of serum TAP in pancreatic acinar cells. All experiments were performed at least three times. Data are presented as Mean ± SEM. *p < 0.05 0.5 h CCK versus 0.5 h normal control (NC), ^#^ *p* < 0.05 1 h CCK versus 1 h NC, ^+^ *p* < 0.05 3 h CCK versus 3 h NC, ^δ^ *p* < 0.05 6 h CCK versus 6 h NC. CCK, cholecystokinin.

It is reported that several pathological events such as ER stress and necroptosis are involved in AP (38). To investigate the relationship of ER stress and necroptosis in AP, pancreatic acinar cells were stimulated by 200 nM CCK *in vitro*, ER stress indicators such as GRP78, sXBP1 and CHOP, and necroptosis markers such as RIP3 and pMLKL were detected by western blotting. Data showed that GRP78, sXBP1 and CHOP were elevated significantly at 0.5 ~1 h (**Figure 1A**); RIP3 and pMLKL were markedly increased at 6 h (**Figure 1B**). That is to say, ER stress was activated in the early stage of CCK stimulation in acinar cells, while necroptosis activated in the late stage. Besides, trypsinogen was activated at 0.5 h (**Figure 1C**). Then we induced ER stress in acinar cells by 2 μM thapsigargin (Tg) for 6 h. As shown in **Supplementary Figure 1**, RIP3 level was increased at 12 ~24 h and MLKL was phosphorylated at 18 ~24 h in a dose-dependent manner. However, ATP depletion was only slightly elevated (about 5 ~10%), and trypsinogen cannot be activated by 2 μM Tg. Therefore, ER stress may regulate necroptosis, independent of trypsinogen activation.

### Inhibition of ER stress alleviated necroptosis during AP

**Figure 2:**
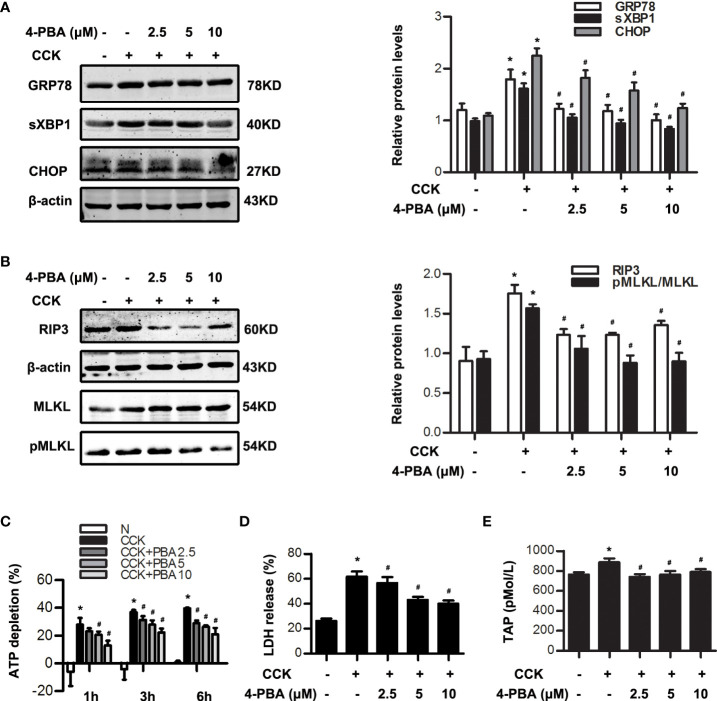
ER stress inhibition reduced necroptosis in CCK -stimulated pancreatic acinar cells. **(A)** Pancreatic acinar cells were pre-treated with ER stress inhibitor 4-PBA (2.5 μM, 5 μM, 10 μM) for 30 min and then stimulated by 200 nM CCK for 0.5 h. Immunoblot analysis of GRP78, sXBP1 and CHOP levels in pancreatic acinar cells. **(B)** Pancreatic acinar cells were pre-treated with ER stress inhibitor 4-PBA (2.5 μM, 5 μM, 10 μM) for 30 min and then stimulated by 200 nM CCK for 6 h. Immunoblot analysis of RIP3, MLKL and pMLKL levels in pancreatic acinar cells. **(C)** Pancreatic acinar cells were pre-treated with ER stress inhibitor 4-PBA (2.5 μM, 5 μM, 10 μM) for 30 min and then stimulated by 200 nM CCK for 1 h, 3 h and 6 h. Cell viability analysis of ATP levels in pancreatic acinar cells. **(D, E)** Pancreatic acinar cells were pre-treated with ER stress inhibitor 4-PBA (2.5 μM, 5 μM, 10 μM) for 30 min and then stimulated by 200 nM CCK for 6 h. **(D)** LDH release analysis of pancreatic acinar cells. **(E)** ELISA of serum TAP in pancreatic acinar cells. All experiments were performed at least three times. Data are presented as Mean ± SEM. *p < 0.05 versus NC, ^#^ *p* < 0.05 versus CCK. CCK, cholecystokinin; 4-PBA, 4-phenylbutyrate.

In order to explore the relationship between ER stress and necroptosis, 4-PBA (2.5 μM, 5 μM, 10 μM), a specific inhibitor of ER stress, was used 30 min in advance and then stimulated by 200 nM CCK *in vitro*. Data indicated that GRP78, sXBP1 and CHOP levels were dose-dependently inhibited by 4-PBA in CCK-stimulated acinar cells (**Figure 2A**). Furthermore, we found that 4-PBA significantly blocked necroptosis in a dose-dependent manner in CCK-stimulated acinar cells *in vitro*, manifested in the decreased RIP3 and pMLKL levels. 4-PBA also significantly reduced CCK-induced ATP depletion, and LDH release rate in CCK-stimulated pancreatic acinar cells (**Figure 2C, D**). In addition, TAP levels were elevated after CCK stimulation, which were remarkably reduced after 4-PBA treatment (**Figure 2E**). These data showed that ER stress inhibition by 4-PBA significantly alleviated pancreatic acinar cell necroptosis during AP *in vitro*.

**Figure 3:**
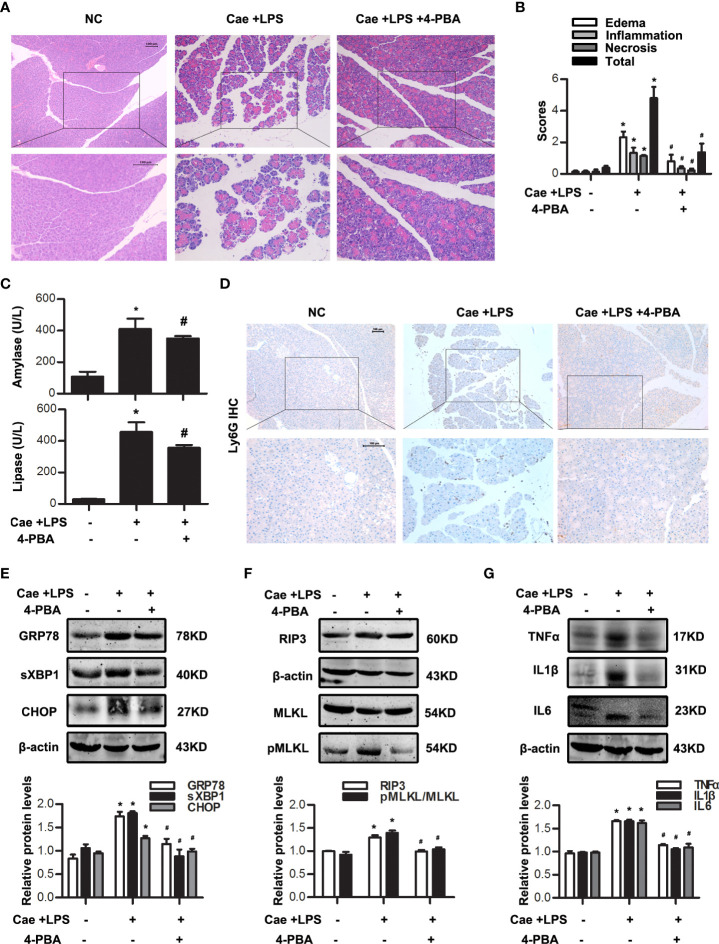
ER stress inhibition alleviated necroptosis in caerulein and LPS-induced AP model. *In vivo*, AP was induced by injection of caerulein (100 μg·kg^-1^) and LPS (5 mg·kg^-1^) in Balb/C mice, and treated with 4-PBA (4mg per mouse, i.p., 0.5 h before the first caerulein injection). **(A)** Representative micrographs of H&E-stained pancreatic sections (200 ×). **(B)** Histological scores were determined as described in Methods. **(C)** Change in serum activity of amylase (up) and lipase (down). **(D)** Representative micrographs of neutrophil marker Ly6G immunohistochemical analyses in pancreas (200 ×). **(E)** Immunoblot analysis of GRP78, sXBP1 and CHOP levels of pancreatic tissue in mice. **(F)** Immunoblot analysis of RIP3, MLKL and pMLKL levels of pancreatic tissue in mice. **(G)** Immunoblot analysis of TNFα, IL1β and IL6 levels of pancreatic tissue in mice. All experiments were performed at least three times. Data are presented as Mean ± SEM. n = 6 per group. Scale bar = 100 μm. *p < 0.05 versus NC, ^#^ *p* < 0.05 versus AP. Cae, caerulein; LPS, lipopolysaccharide; NC, normal control; 4-PBA, 4-phenylbutyrate.

**Figure 4:**
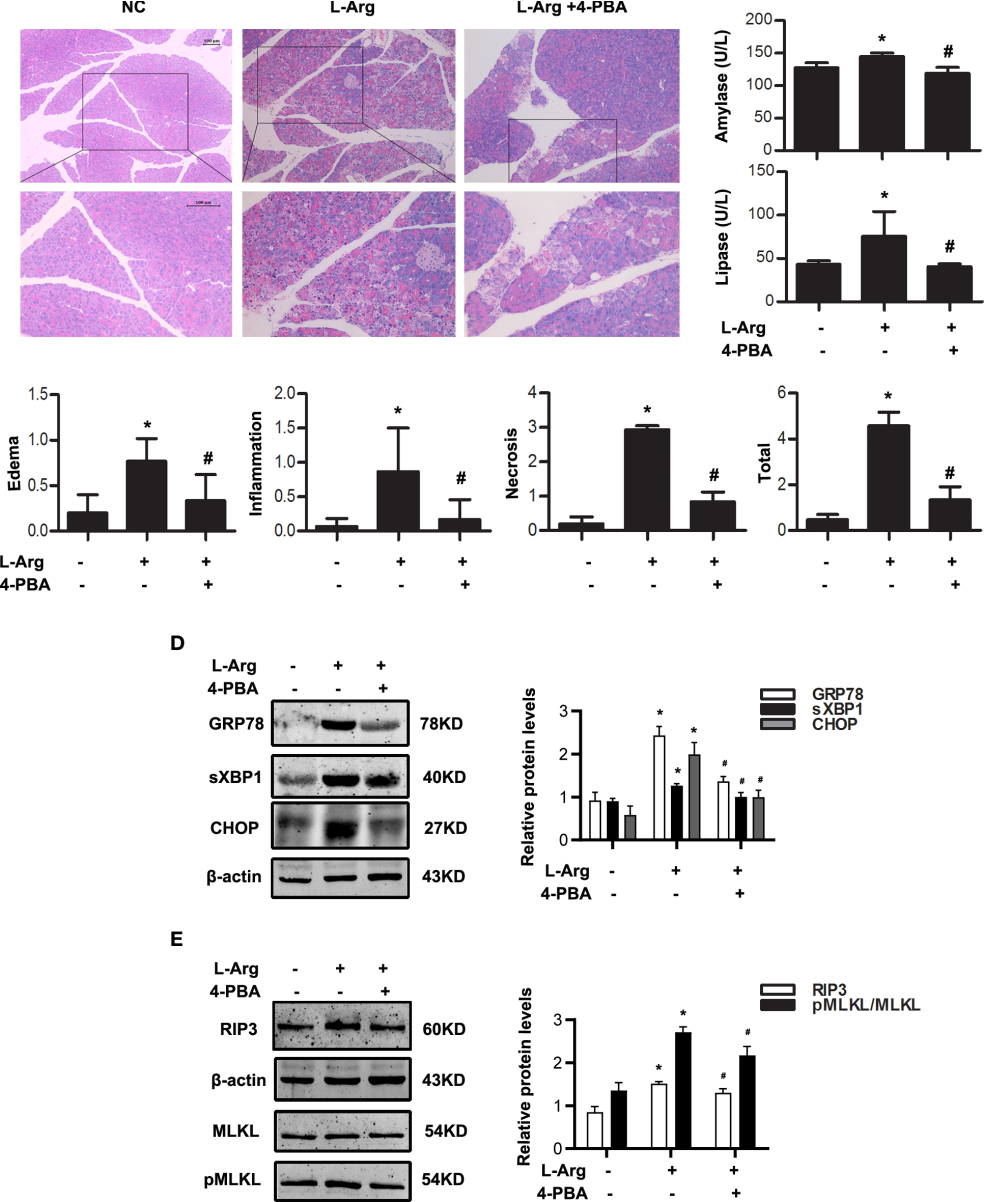
ER stress inhibition alleviated necroptosis in L-arginine-induced AP model. *In vivo*, AP was induced by injection of L-arginine (10 mg·kg^-1^) in Balb/C mice, and treated with 4-PBA (4mg per mouse, i.p., 0.5h before the first L-arginine injection). **(A)** Representative micrographs of H&E-stained pancreatic sections (200 ×). **(B)** Histological scores were determined as described in Methods. **(C)** Change in serum activity of amylase (up) and lipase (down). **(D)** Immunoblot analysis of GRP78, sXBP1 and CHOP levels of pancreatic tissue in mice. **(E)** Immunoblot analysis of RIP3, MLKL and pMLKL levels of pancreatic tissue in mice. All experiments were performed at least three times. Data are presented as Mean ± SEM. n = 6 per group. Scale bar = 100 μm. *p < 0.05 versus NC, ^#^ *p* < 0.05 versus AP. L-Arg, L-arginine; NC, normal control; 4-PBA, 4-phenylbutyrate.

*In vivo*, caerulein and LPS-induced AP model in Balb/C mice were built with or without 4-PBA treatment at 0.5 h in advance. Histological examination showed that the extent of pancreatic injury in 4-PBA-treated mice was less severe than that in AP group at 12 h after the first caerulein injection (**Figure 3A, B** **)**. Amylase and lipase activities were markedly decreased in the sera of 4-PBA-treated mice compared to AP groups (**Figure 3C**). Furthermore, we examined the infiltration of Ly6G^+^ neutrophils in the pancreatic tissue by immunohistochemistry staining. Data showed that 4-PBA reduced Ly6G^+^ neutrophil infiltration in pancreas when compared to AP groups (**Figure 3D**). 4-PBA treatment blocked the upregulation of ER stress indicators such as GRP78, sXBP1 and CHOP, and necroptosis markers such as RIP3 and pMLKL during AP (**Figure 3E, F**). In addition, the serum TNFα, IL1β and IL6 levels were also markedly decreased in 4-PBA pre-treated mice compared to AP group (**Figure 3G**). L-Arg-induced AP model were also built with or without 4-PBA treatment. The similar results were obtained at day 3 after the second L-Arg injection, as shown in **Figure 4**. We found that 4-PBA significantly alleviated pancreatic injury assessing by histological examination and pathological score analysis; reduced serum amylase and lipase levels; and markedly decreased GRP78, sXBP1, CHOP, RIP3 and pMLKL levels in pancreatic tissue during L-Arg-induced AP (**Figure 4A-E**). Collectively, these results suggested that inhibition of ER stress by 4-PBA alleviated pancreatic necroptosis during AP both *in vitro* and *in vivo*.

### ER stress motivated CTSB maturation and AP-1 activation *via* PKCα-JNK-cJun pathway

**Figure 5:**
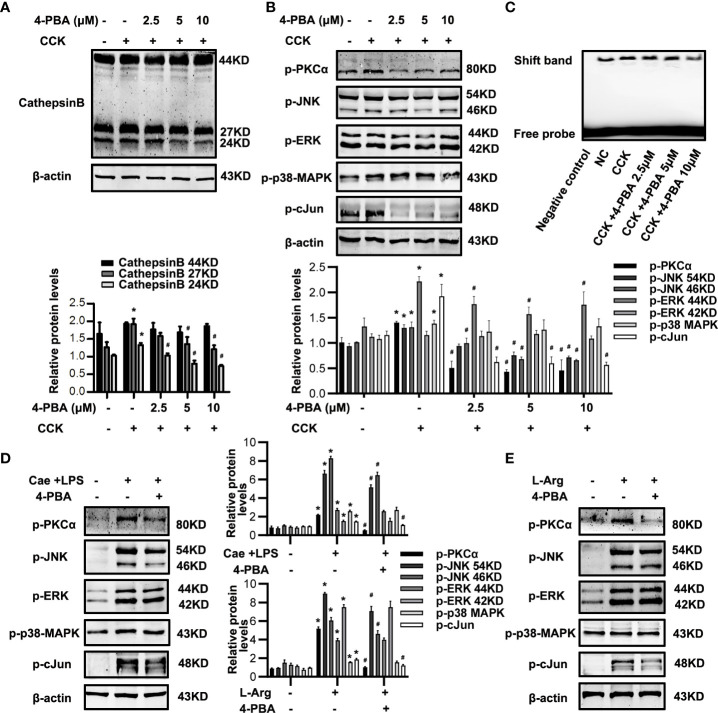
ER stress inhibition alleviated CTSB maturation and PKCα-JNK-cJun-mediated AP-1 activation in AP. **(A-C)** *In vitro*, Pancreatic acinar cells were pre-treated with ER stress inhibitor 4-PBA (2.5 μM, 5 μM, 10 μM) for 30 min and then stimulated by 200 nM CCK for 6 h. **(A)** Immunoblot analysis of CTSB levels in pancreatic acinar cells. **(B)** Immunoblot analysis of p-PKCα, p-JNK, p-ERK, p-p38-MAPK and p-cJun levels in pancreatic acinar cells. **(C)** EMSA analysis of AP-1 binding ability in pancreatic acinar cells. **(D)** *In vivo*, AP was induced by injection of caerulein (100 μg·kg^-1^) and LPS (5 mg·kg^-1^) in Balb/C mice, and treated with 4-PBA (4 mg per mouse, i.p., 0.5 h before the first caerulein injection). Immunoblot analysis of p-PKCα, p-JNK, p-ERK, p-p38-MAPK and p-cJun levels of pancreatic tissue in mice. **(E)** *In vivo*, AP was induced by injection of L-arginine (10 mg·kg^-1^) in Balb/C mice, and treated with 4-PBA (4 mg per mouse, i.p., 0.5 h before the first L-arginine injection). Immunoblot analysis of p-PKCα, p-JNK, p-ERK, p-p38-MAPK and p-cJun levels of pancreatic tissue in mice. All experiments were performed at least three times. Data are presented as Mean ± SEM. n = 6 per group. *p < 0.05 versus NC, ^#^ *p* < 0.05 versus CCK or AP. Cae, caerulein; CCK, cholecystokinin; CTSB, cathepsin B; LPS, lipopolysaccharid; L-Arg, L-arginine; NC, normal control.

The detailed mechanisms of ER stress mediating necroptosis remains unclear. CTSB is well-known as a protease to activate intrapancreatic trypsinogen and initiate the onset of AP (21, 23). Recent studies suggest that excessive CTSB released from lysosomes into the cytosol can shift the cell death pathway towards necroptosis (23). TNFα is a ligand binding to death receptor to motivate necroptosis, and AP-1 is a transcription factor encoding the expression of inflammatory factors, such as TNFα (25, 26). To determine the detailed mechanisms of ER stress mediating necroptosis, we investigated the role of CTSB maturation and AP-1 activation during ER stress both in pancreatic acinar cells *in vitro* and in pancreatic tissue of experimental AP model *in vivo*. Firstly, western blotting analysis showed that 4-PBA significantly reduced the expression of mature CTSB in a dose-dependent manner in CCK-stimulated acinar cells *in vitro* (**Figure 5A**). Secondly, phosphorylation levels of PKCα, JNK and cJun were markedly decreased by 4-PBA, while ERK and p38MAPK phosphorylation levels showed no significant difference after 4-PBA treatment in CCK-stimulated acinar cells *in vitro* (**Figure 5B**). Next, further experiments showed that 4-PBA then reduced AP-1 binding activity in CCK-stimulated acinar cells (**Figure 5C**), as previous studies have shown that PKC-MAPKs signaling pathway can activate AP-1 (24). Lastly, we confirmed these results both in caerulein and LPS-induced and L-Arg-induced AP models *in vivo*. Consistent with the results *in vitro*, we also found that 4-PBA significantly inhibited the phosphorylation levels of PKCα, JNK and cJun, but not ERK and p38MAPK phosphorylation levels both in two experimental AP models *in vivo* (**Figure 5D, E**). To sum up, ER stress blockade by 4-PBA not only inhibited CTSB maturation but also suppressed AP-1 activation *via* PKCα-JNK-cJun pathway during AP both *in vitro* and *in vivo*.

### CTSB induced AP-1 activation and necroptosis *via* PKCα-JNK-cJun pathway in AP independent of trypsin activation

**Figure 6:**
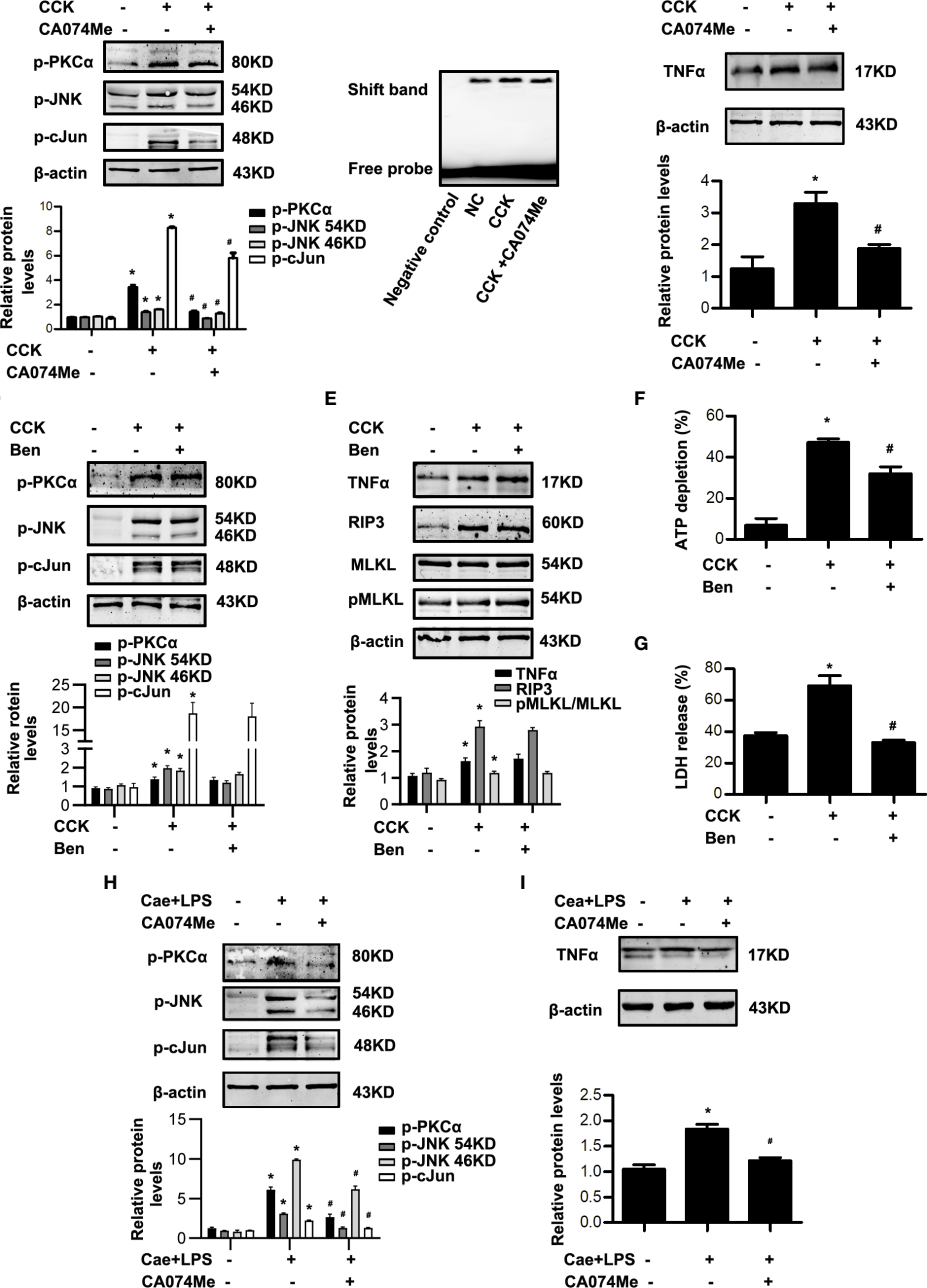
CTSB inhibitor CA074Me blocked PKCα-JNK-cJun-mediated AP-1 activation in AP, which had no concern with trypsin. **(A-C)** *In vitro*, pancreatic acinar cells were pre-treated with ER stress inhibitor CA074Me (50 μM) for 30 min and then stimulated by 200 nM CCK for 30 min or 6 h. **(A)** Immunoblot analysis of p-PKCα, p-JNK, and p-cJun levels in pancreatic acinar cells. **(B)** EMSA analysis of AP-1 binding ability in pancreatic acinar cells. **(C)** Immunoblot analysis of TNFα levels of pancreatic acinar cells. **(D-G)** *In vitro*, pancreatic acinar cells were pre-treated with trypsin inhibitor benzamidine hydrochloride (Ben; 1 μM) and stimulated by 200 nM CCK for 30 min or 6 h. **(D)** Immunoblot analysis of p-PKCα, p-JNK, and p-cJun levels in pancreatic acinar cells. **(E)** Immunoblot analysis of TNFα, RIP3, MLKL and pMLKL levels in pancreatic acinar cells. **(F)** Cell viability analysis of ATP levels in pancreatic acinar cells. **(G)** LDH release analysis of pancreatic acinar cells. **(H, I)** *In vivo*, AP was induced by injection of caerulein (100 μg·kg^-1^) and LPS (5 mg·kg^-1^) in Balb/C mice, and treated with CA074Me (10 mg/kg, i.p., 0.5h before the first caerulein injection. **(H)** Immunoblot analysis of p-PKCα, p-JNK and p-cJun levels of pancreatic tissue in mice. **(I)** Immunoblot analysis of TNFα levels of pancreatic tissue in mice. All experiments were performed at least three times. Data are presented as Mean ± SEM. n = 6 per group. *p < 0.05 versus NC, ^#^ *p* < 0.05 versus CCK or AP. Ben, benzamidine hydrochloride; CA074Me, CA074 Methyl ester; Cae, caerulein; CCK, cholecystokinin; LPS, lipopolysaccharid; NC, normal control.

Next, in order to clarify the relationship between CTSB and AP-1, CTSB inhibitor CA074Me was administered to block CTSB at 30 min before caerulein and LPS or CCK challenge, and then AP-1 activation was determined. *In vitro*, blockade of CTSB by CA074Me significantly inhibited the phosphorylation levels of PKCα, JNK and cJun, AP-1 activation and TNFα levels in CCK-stimulated pancreatic acinar cells (**Figure 6A-C**). It was reported that CTSB can activate trypsinogen (21, 23). In order to exclude the influence of trypsinogen activation, we used benzamidine hydrochloride to inhibit trypsin activity and observed changes of PKCα-JNK-cJun pathway, TNFα level and necroptosis. Notably, our data showed that benzamidine hydrochloride had no effect on phosphorylation levels of PKCα, JNK and cJun, TNFα levels, or necroptosis markers including RIP3 and pMLKL in CCK-stimulated acinar cells (**Figure 6D, E**), although total ATP depletion and LDH release rate significantly reduced after benzamidine hydrochloride treatment (**Figure 6F, G**). In summary, CTSB participated in AP through two pathways: one is to activate AP-1 *via* PKCα-JNK-cJun signaling and induce necroptosis, which is independent of trypsin activity; and the other way is to activate trypsinogen and induce necrosis. Similarly, we observed the effect of CA074Me on caerulein and LPS-induced AP model *in vivo*. As expected, CA074Me led to a significant decrease of PKCα, JNK, cJun phosphorylation levels and TNFα levels in pancreatic tissue (**Figure 6H, I**). That is to say, CTSB enhanced AP-1 activation and TNFα levels to induce necroptosis *via* PKCα-JNK-cJun pathway in caerulein and LPS-induced AP model. In a word, CTSB induced AP-1 activation and necroptosis *via* PKCα-JNK-cJun pathway during AP, independent of trypsin activation.

### AP-1 induced necroptosis of acinar cells *via* TNFα autocrine secretion in AP

**Figure 7:**
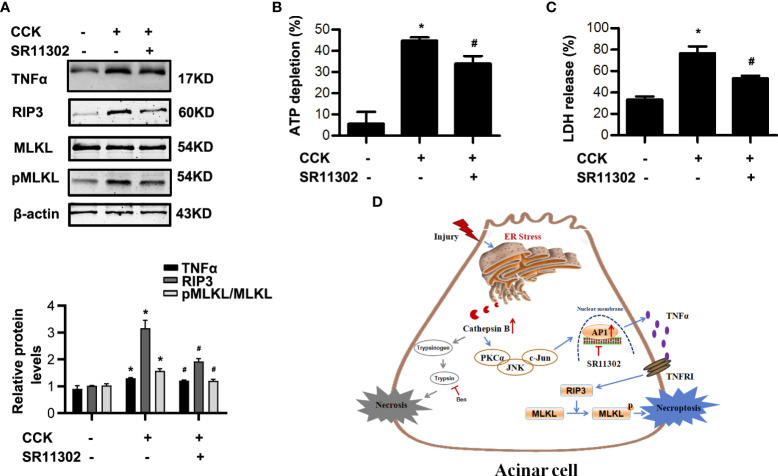
AP-1 inhibitor SR11302 restrained necroptosis in CCK-stimulated pancreatic acinar cells. Acinar cells were pre-treated with AP-1 inhibitor SR11302 (10 μM) for 30 min, and then stimulated by 200 nM cholecystokinin (CCK) 8 for 6 h. **(A)** Immunoblot analysis of RIP3, MLKL and pMLKL levels in pancreatic acinar cells. **(B)** Cell viability analysis of ATP levels in pancreatic acinar cells. **(C)** LDH release analysis of pancreatic acinar cells. **(D)** Schematic diagram summarizing the mechanisms by which ER stress influences necroptosis in experimental pancreatitis. Endoplasmic reticulum stress promoted acinar cell necroptosis through cathepsin B -induced AP-1 activation and TNFα autocrine secretion in acute pancreatitis. All experiments were performed at least three times. Data are presented as Mean ± SEM. *p < 0.05 versus NC, ^#^ *p* < 0.05 versus CCK. CCK, cholecystokinin.

AP-1, as an important transcription factor, is related to the transcription of TNFα (24, 26), which has been demonstrated to trigger necroptosis in many cell types (6, 7). In order to determine the role of AP-1 on necroptosis during AP, we used SR11302 30 min in advance to inhibit AP-1 in CCK-stimulated pancreatic acinar cells. We found that SR11302 significantly lowered the TNFα, RIP3 and pMLKL levels (**Figure 7A**). Furthermore, treatment with SR11302 also resulted in reduction of CCK-induced pancreatic acinar cells necrosis, manifested as ATP depletion and LDH release rate were reduced markedly by SR11302 in CCK-stimulated pancreatic acinar cells (**Figure 7B, C**). This phenomenon indicated that AP-1 caused acinar cell necroptosis by promoting TNFα autocrine secretion. Taken together, all these results demonstrated that ER stress promoted pancreatic acinar cell necroptosis through CTSB maturation, thus induced AP-1 activation and TNFα autocrine secretion *via* PKCα-JNK-cJun pathway during AP, not related with trypsin activity (**Figure 7D**).

## Discussion

The pathophysiological mechanisms of AP have not been fully elucidated. Pancreas autodigestion caused by trypsinogen activation is considered to be an important event of AP onset, then leads to acinar cell death and inflammation (21). During AP, CTSB can release into the cytosol and cleave trypsinogen to mature trypsin and its NH_2_-terminal trypsinogen activating peptide (TAP), leading to cell death through necrosis (23). Necroptosis is a new type of programmed necrosis discovered in recent years and dependent on RIP3-MLKL pathway (39). Inhibition or gene knockout of RIP3 or MLKL ameliorates experimental AP significantly (6, 7, 10). But the detailed mechanisms of necroptosis regulation need further to be clarified.

ER is responsible for the synthesis, maturation, fold and transport of both secretory and transmembrane proteins, lipid synthesis, carbohydrate metabolism, and calcium storage, which is a dense dynamic, membrane-bounded tubular network organelle (40). Many genetic or environmental factors such as hypoxia, nutrition deficiency, and microbial infection impede ER’s function and lead to the accumulation of misfolded proteins in the ER, which condition is called ER stress or unfolded protein response (UPR) (41). It is reported that ER stress is closely related to apoptosis or necroptosis (19, 20, 40). Imbalance of apoptosis and necrosis plays an important role in AP severity (10, 19, 20). Studies also indicated that ER stress is a contributor to the progression of AP (13); inhibition of ER stress by 4-phenylbutyric acid prevented vital organ injury and intestinal epithelial cell apoptosis in rats with AP, perhaps these effects were involved in alleviating inflammatory response and cell death (16, 17). Therefore, ER stress might influence AP *via* necroptosis. ER changes took place very early in the development of AP in several experimental models (42). Firstly, our data also showed that in CCK-stimulated pancreatic acinar cells, ER stress was initiated minutes after stimulation, which was before necroptosis. Secondly, ER stress induced by Tg in acinar cells increased RIP3 and pMLKL levels in a dose-dependent manner. Lastly, the inhibition of ER stress by 4-PBA can significantly alleviated AP severity *via* reducing necroptosis of acinar cells both in CCK-stimulated pancreatic acinar cell *in vitro* and in caerulein and LPS-induced and L-Arg-induced AP models *in vivo*. These results indicated that ER stress promoted pancreatic acinar cell necroptosis in AP.

Based on relevant literature, CTSB acts as a bridge between ER stress and necroptosis: ER stress activated CTSB in isolated pancreatic acinar cells during AP (22); and excessive CTSB led to necroptosis in tumor cells, by processing of Bid to attack mitochondria (23). The MAPK signalling network is known to regulate cell cycle progression and cell survival or death responses following a variety of stresses; ER stress has been shown to activate JNK and related with cell damage and death (43). Our data also showed that ER stress inhibition significantly reduced the expression of mature CTSB in a dose-dependent manner in CCK-stimulated acinar cells, and ER stress inhibition markedly decreased phosphorylation levels of PKCα, JNK and cJun, rather than ERK and p38MAPK both *in vitro* and *in vivo*. Previous studies have shown that PKC-MAPKs signaling pathway can activate AP-1 (24). Then we used CTSB inhibitor to clarify the relationship between CTSB and AP-1, and used AP-1 inhibitor to indicate the mechanisms of AP-1 regulating necroptosis. We found that CTSB promoted PKCα-JNK-cJun-mediated AP-1 activation and TNFα autocrine, thus causing necroptosis. As is well-known, CTSB is responsible for the trypsinogen activation and AP onset. CTSB^-/-^ significantly reduced trypsin activity and improved the acinar cell necrosis (21). Did CTSB affect necroptosis through trypsin? Our results showed that neither AP-1 nor necroptosis was affected by trypsin inhibitor. Therefore, CTSB induced PKCα-JNK-cJun-mediated AP-1 activation and necroptosis in AP, independent of trypsin activity. Zhang et al. also discovered that CTSB leads to necroptosis in tumor cells, by processing of Bid to attack mitochondria (44); while in McComb’s study, CTSB limits macrophage necroptosis, through cleavage of RIP1 (45). Therefore, the mechanisms of necroptosis may vary slightly among different cell types. In addition, our previous research found that during AP, RIP1 is negatively related to acinar cell necroptosis (10), but the detailed roles of RIP1 in AP and the detailed regulatory mechanisms remain to be investigated.

## Conclusion

In conclusion, our data showed the ability of ER stress to stimulate acinar cell necroptosis *via* CTSB during AP. It is well-known that CTSB can trigger trypsinogen activation and lead to acinar cell necrosis on the one hand; and on the other hand, we found that CTSB can activate AP-1 *via* PKCα-JNK-cJun pathway and induce TNFα-mediated necroptosis of pancreatic acinar cells. Therefore, the finding that ER stress induced necroptosis *via* CTSB may provide potential new targets and treatment strategies of AP or other cell death-related diseases.

## Data availability statement

The original contributions presented in the study are included in the article/**Supplementary Material**. Further inquiries can be directed to the corresponding authors.

## Ethics statement

This study was reviewed and approved by Animal Ethics Committee of Shanghai Jiao Tong University School of Medicine (SYXK 2013-0050, Shanghai, China).

## Author contributions

XH performed all the experiments, analyzed data and drafted the manuscript. BL, JB and JW performed the experiments and analyzed data. ZW, PS, QP, CC, JN and JS, provided technical support in the *in vivo* and *in vitro* experiments. GH designed, conceived the study, analyzed data, and revised the manuscript. XH, GH, and JW provided funding to support the study. GH, XW and RW supervised the study. All authors contributed to the article and approved the submitted version.

## Funding

This work was sponsored by National Natural Science Foundation of China to XH (81900584), GH (81670584, 81970556 and 82170652), and JW (81800568), Shanghai Sailing Program to XH (19YF1438900).

## Conflict of interest

The authors declare that the research was conducted in the absence of any commercial or financial relationships that could be construed as a potential conflict of interest.

## Publisher’s note

All claims expressed in this article are solely those of the authors and do not necessarily represent those of their affiliated organizations, or those of the publisher, the editors and the reviewers. Any product that may be evaluated in this article, or claim that may be made by its manufacturer, is not guaranteed or endorsed by the publisher.
