# Supplementary material for: Endoplasmic reticulum stress promoted acinar cell necroptosis in acute pancreatitis through cathepsinB-mediated AP-1 activation
Source: Front Immunol. 2022 Aug 19;13:968639. doi: 10.3389/fimmu.2022.968639 (PMC9438943; doi:10.3389/fimmu.2022.968639)
Supplement: Supplementary file 1 [file DataSheet_1.docx]

Supplementary Material

## The Supplementary material contains one Supplementary Figure and Figure Legend.

## Supplementary Figure


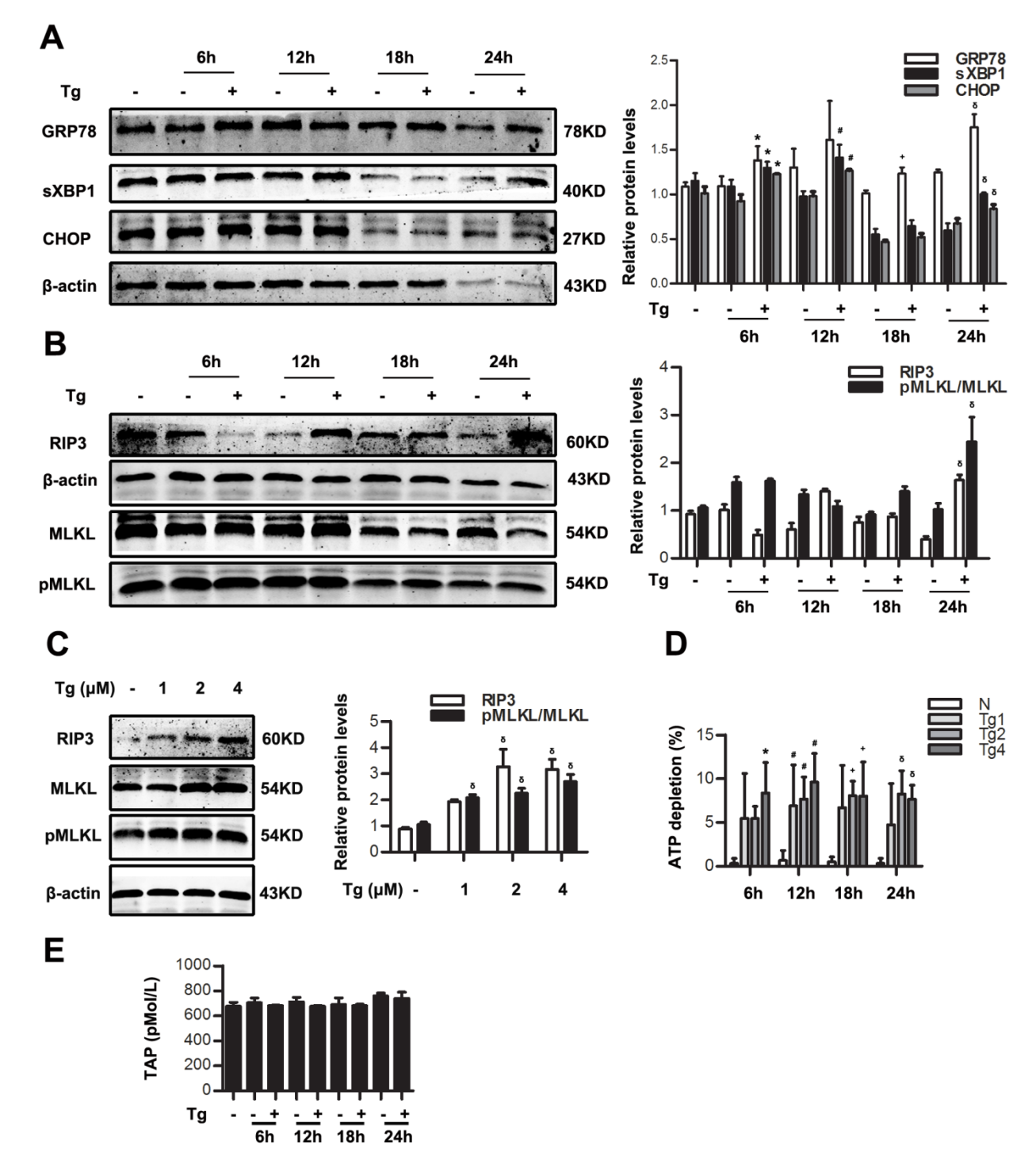


**Supplementary Figure 1.** Pancreatic acinar cells were stimulated by 1, 2 or 4 μM thapsigargin (Tg) for 6 h, 12 h, 18 h and 24 h. **(A)** Immunoblot analysis of GRP78, sXBP1 and CHOP levels in pancreatic acinar cells. **(B)** Immunoblot analysis of RIP3, MLKL and pMLKL levels in pancreatic acinar cells. **(C)** Immunoblot analysis of RIP3, MLKL and pMLKL levels in pancreatic acinar cells at 24h. **(D)** Cell viability analysis of ATP levels in pancreatic acinar cells. All experiments were performed at least three times. Data are presented as Mean ±SEM. **p* < 0.05 6 h CCK versus 6 h NC, ^#^*p* < 0.05 12 h CCK versus 12 h NC, ^+^*p* < 0.05 18 h CCK versus 18 h NC, ^δ^*p* < 0.05 24 h CCK versus 24 h NC.
